# Occurrence and Genetic Characterization of Grapevine Pinot Gris Virus in Russia

**DOI:** 10.3390/plants11081061

**Published:** 2022-04-13

**Authors:** Darya Shvets, Svetlana Vinogradova

**Affiliations:** Institute of Bioengineering, Research Center of Biotechnology of the Russian Academy of Sciences, Leninsky Prospect 33, 119071 Moscow, Russia; darya-shv@mail.ru

**Keywords:** Grapevine Pinot gris virus, *Trichovirus*, viral diagnostics, real-time PCR, phylogenetic analysis, single-nucleotide polymorphisms, post-translational modifications

## Abstract

Grapevine Pinot gris virus (GPGV) is a widespread grapevine pathogen associated with symptoms of leaf mottling and deformation. In order to study the distribution and genetic diversity of GPGV in Russia, we tested 1347 grapevine samples from 3 regions of Russia–the Krasnodar Krai, Stavropol Krai, and Republic of Crimea—using duplex real-time RT-PCR. GPGV was detected in 993 grapevines, both symptomatic and asymptomatic. In 119 isolates, we sequenced complete movement protein (MP) and coat protein (CP) genes of the GPGV genome. The percentage of identity of the obtained nucleotide MP/CP sequences with the closest isolates from the GenBank was 97.75–99.56%. A phylogenetic analysis showed that these Russian GPGV isolates are mainly grouped with previously described representative asymptomatic isolates. New post-translational modifications of the MP and CP at the positions of polymorphisms in the genomes of Russian isolates were predicted. The present work is the first study on the distribution and genetic diversity of GPGV in Russia.

## 1. Introduction

Grapevine Pinot gris virus (GPGV) is a member of the genus *Trichovirus* of the family Betaflexiviridae. In 2012, it was identified for the first time on a grapevine using the high-throughput sequencing (HTS) method [1].

The GPGV genome is represented by a linear (+) ssRNA molecule consisting of three overlapping open reading frames (ORFs): ORF1 encodes the replicase-associated proteins, and ORF2 and ORF3 encode the movement protein (MP) and coat protein (CP), respectively [1,2].

The presence of GPGV has been reported in most grape growing countries: Italy [1], Korea [3], Slovakia [4], Czech Republic [4], Slovenia [5], Uruguay [6], Georgia [7], France [8], Switzerland [9], Canada [10], Germany [11], Turkey [12], the USA [13], China [14], Bosnia and Herzegovina [15], Croatia [15], Montenegro [15], North Macedonia [15], Portugal [15], Romania [15], Serbia [15], Ukraine [15], Spain [15], Brazil [16], Australia [17], Poland [18], Pakistan [19], Great Britain [20], Hungary [21], Lebanon [22], Moldova [23], Chile [24], Argentina [25], Armenia [26], Iran [27], Belgium [28], Algeria [29], Japan [30], and Bulgaria [31]. Previously, we detected GPGV in the vineyards of the Krasnodar Krai using the HTS method [32]. At the same time, data indicating the presence of GPGV in other viticultural regions of Russia did not exist.

In some cultivars and rootstocks of grapes, the presence of GPGV correlates with grapevine leaf mottling and deformation (GLMD) disease [33]. There are several forms of GLMD manifestation: mild, moderate, and severe [34,35]. Moreover, GPGV infection may be asymptomatic [1]. Therefore, the relationship between the presence of the virus and the manifestation of symptoms remains unclear [4,36,37,38].

Several studies suggested that the presence of symptoms depends on the genetic variability of GPGV isolates [38,39,40,41,42]. It has been observed that symptomatic isolates containing T at position 6685 of the GPGV genome in the MP gene, which leads to premature termination of translation, are grouped into separate genetic clades from asymptomatic ones [36]. However, the correlation of symptoms with the presence or absence of the polymorphism at position 6685 of the genome sequence is not always observed [39,40]. This may indicate the presence of other variations in the GPGV nucleotide sequence that determine the manifestation of GLMD disease. Nonetheless, the role of sequence variation at the C-terminus of the MP gene of GPGV in the manifestation of symptoms in plants has been reliably confirmed [43]. Moreover, the manifestation of symptoms can correlate not only with different genetic variants of GPGV but also with virus titer: there is evidence that symptomatic grapevines have a higher GPGV titer compared to asymptomatic grapevines [37,39,43,44]. However, the study by Morán et al. (2018) did not confirm such a correlation [41].

The aim of the present work was to study the distribution and genetic diversity of GPGV in Russia. We detected GPGV in all major viticultural regions of Russia: the Krasnodar Krai, Stavropol Krai, and Republic of Crimea. Analysis of the MP/CP region of the GPGV genome revealed 469 polymorphisms, including those leading to nonsynonymous amino acid substitutions, most of which were located at the C-terminus of MP. New sites of post-translational modifications of MP and CP at the positions of polymorphisms of Russian isolates were predicted. This work contributes to the study of the distribution and epidemiology of GPGV in Russia.

## 2. Results

### 2.1. Determination of qPCR Parameters

The real-time quantitative RT-PCR (qRT-PCR) parameters for the target CP gene and internal control genes *Cpn*21 (chaperonin 21) and *GAPDH* (glyceraldehyde-3-phosphate dehydrogenase) in simplex and duplex assays are provided in Table 1. The R^2^ value for all genes exceeded 0.96, which is close to 1 and corresponds to the optimal theoretical value [45]. The values of the efficiency for the target CP gene and the internal control gene of chaperonin were 96% and 102% in the simplex assay and 97% and 94% in the duplex assay, respectively, which is within the optimal range of 90–110% [46]. The slope value for the CP and *Cpn*21 genes in the simplex and duplex assays corresponded to the theoretically acceptable range of −3.58 to −3.10 [47]. In addition, the mean Cq values did not differ for CP detected in the simplex assay and CP/*Cpn*21 detected in the duplex assay for the dilutions shown (Supplementary Table S1). Thus, the presence of primers and probes for the chaperonin gene did not affect the sensitivity of the target reaction. At the same time, the qPCR efficiency for the internal control gene *GAPDH* was 74% for the simplex assay and 78% for the duplex assays, and the slope of the standard curve was below −3.6, which did not correspond with the optimal values and indicates the low assay sensitivity of the CP/*GAPDH* duplex. Moreover, the mean Cq values were statistically different for CP in the simplex assay and CP/*GAPDH* in the duplex assay (Supplementary Table S1). Therefore, the use of *GAPDH* as part of the duplex affects the sensitivity of the target reaction. Thus, based on the obtained parameters characterizing qPCR, we chose the CP/*Cpn*21 duplex for the detection of GPGV during phytosanitary monitoring.

### 2.2. Monitoring of GPGV in Vineyards of Russia

**Figure 1:**
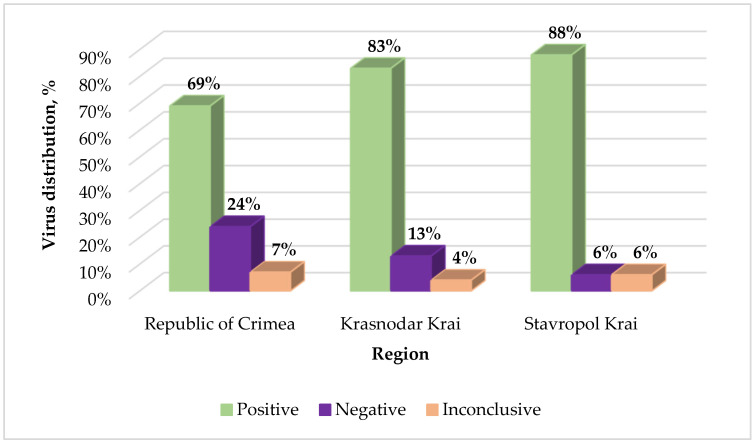
Graphical representation of Grapevine Pinot gris virus monitoring in various regions of Russia. Percentages were calculated from the total number of samples collected in each region.

Examination of Russian vineyards by qRT-PCR showed that in the Republic of Crimea, 634 out of the total 918 samples (69% of the total number of samples collected in this region) were positive for GPGV and 217 samples (24%) were negative (Figure 1). GPGV was detected in 330 out of the total 396 samples (83%) from the Krasnodar Krai while 52 grapevines (13%) showed negative testing results. In the Stavropol Krai, the presence of GPGV was shown in 29 out of the total 33 samples (88%); in 2 samples (6%), the virus was not found. In total, GPGV was found in 993 samples in the 3 regions of Russia, representing the vast majority of vineyards. In general, the relative ratio values varied in different samples, ranging from 2.74 × 10^−6^ to 6.12 (Supplementary Table S2). However, in some samples (67 vines in the Republic of Crimea, 14 vines in the Krasnodar Krai, and 2 vines in the Stavropol Krai), the Cq values for the CP gene were determined only in one of two replicates in each experiment. Repeated qPCR for such samples usually did not lead to a change in the result. These samples with unstable detection were designated as inconclusive.

### 2.3. Phylogenetic Analysis

For 119 Russian isolates of GPGV, we determined 1600 bp nucleotide sequences of the MP/CP region. The percentage of identity at the nucleotide level with the closest isolates from the GenBank was 97.75–99.56% (Supplementary Table S3). No recombination events were detected in Russian GPGV isolates.

**Figure 2:**
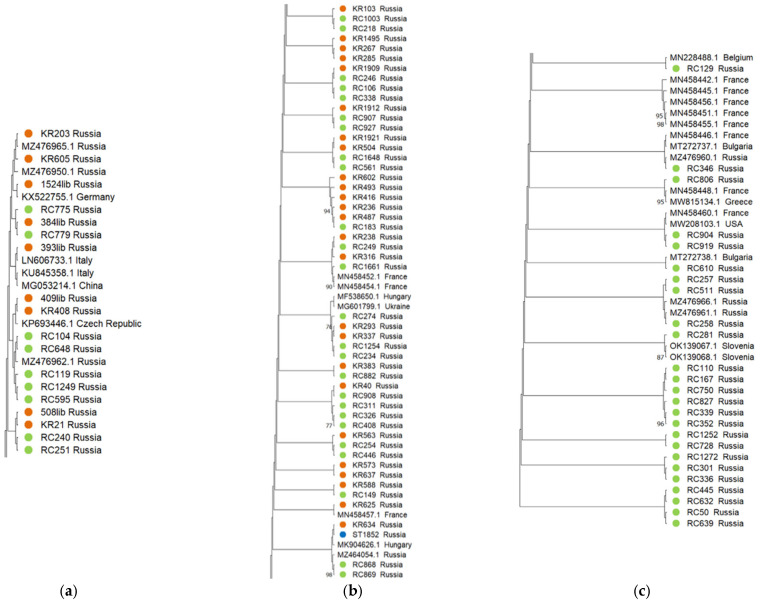
Phylogenetic analysis of global isolates of GPGV based on nucleotide sequences of the movement protein (MP) and coat protein (CP) genes. Russian isolates from the Krasnodar Krai, Stavropol Krai, and Republic of Crimea are marked in orange, blue, and green, respectively. The main clades with the largest number of Russian isolates are shown in (**a**–**c**). The tree was constructed using the maximum likelihood method. The numbers at the nodes indicate bootstrap support (1000 replicates); values above 60% are shown. Branches corresponding to partitions reproduced in less than 50% of the bootstrap replicates are collapsed.

A phylogenetic analysis of Russian and world isolates of GPGV showed that most of the isolates identified by us grouped into three clades. The first clade, in addition to Russian isolates, included isolates from Germany, China, Italy, and the Czech Republic (Figure 2a). The second clade included isolates from France, Hungary, and Ukraine (Figure 2b), and the third clade included isolates from Belgium, France, Bulgaria, Greece, the USA, and Slovenia (Figure 2c). It should be noted that the first two clades comprised Russian isolates from the Republic of Crimea, Krasnodar Krai, and Stavropol Krai, whereas the third clade included isolates only from the Republic of Crimea. Moreover, individual Russian isolates clustered with isolates from Italy, Hungary, China, Slovenia, Slovakia, Canada, Turkey, Ukraine, and Pakistan.

### 2.4. Variability of the GPGV Genome

A comparison of the MP/CP nucleotide sequences in the genomes of Russian isolates showed that the pairwise identity varied from 95.7% to 100% (Supplementary Table S4 and Figure S1a), with isolates with identity scores ranging from 97.0–98.9% predominating (Supplementary Table S5). Two isolates (RC868 and RC869 collected from the same vineyard but from different cultivars: Pinot Noir and Chardonnay, respectively) were 100% identical to each other. We also identified a group of isolates (RC281, RC282, RC287, RC288, RC290, KR130, KR267, KR281, KR285, and KR383) that were most different from other Russian isolates (highlighted in blue in Supplementary Figure S1a). The average value of the pairwise identity for them was between 96.66 and 97.43%, whereas the average value of the pairwise identity for the other isolates was between 97.70 and 98.81%. On the phylogenetic tree, divergent Russian GPGV isolates closely clustered with each other, with the exception of 1 isolate KR383, the sequence of which was 97.4% similar to that of the RC882 isolate (Supplementary Figure S2).

For the amino acid sequences of the MP of the Russian isolates, the pairwise identity score ranged from 94.9 to 100% (Supplementary Table S6 and Figure S1b). The most common identity scores were 97.0–99.9% (Supplementary Table S5). By identity score, the biggest differences were found for the amino acid sequences of the RC282, RC287, RC288, RC728, KR130, KR267, KR281, KR285, KR408, and KR1495 isolates, the mean identity score for which ranged from 96.36 to 97.51%, whereas the mean identity score for most other isolates was between 97.59 and 99.07%.

For the amino acid sequences of the CP of the Russian isolates, the identity score was 96.4–100% (Supplementary Table S7 and Figure S1c); most of the isolates had identity scores of 99.0–100% (Supplementary Table S5). The RC119 isolate from the Republic of Crimea (cultivar of Rkatsiteli) had the least similarity with other isolates in the CP sequence (the mean identity scores were 97.34%).

**Figure 3:**
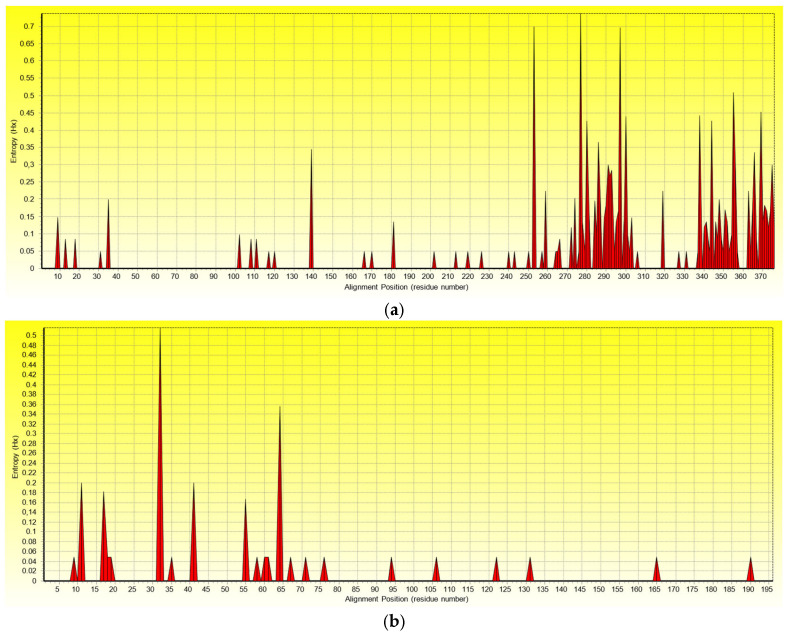
Graphical representation of the entropy of the (**a**) movement and (**b**) coat protein sequences generated using the entropy plot function (H(x)). The plot shows the variability of the different amino acid residues in alignment with Russian isolates of GPGV.

A comparison of the nucleotide sequences of the Russian GPGV isolates showed the presence of 469 single-nucleotide polymorphisms (SNPs) in the MP/CP region that lead to transitions and transversions. Nucleotide variability was uniformly distributed throughout the MP/CP genes of GPGV (Supplementary Figure S3). In total, 88 SNPs in the MP gene and 22 in the CP gene resulted in nonsynonymous substitutions. An entropy plot analysis of the amino acid sequences showed that a larger number of amino acid substitutions were observed at the 3’ end of MP and the 5’ end of CP (Figure 3).

As a result of the analysis of non-synonymous amino acid substitutions, the presence of 23 post-translational modifications (PTMs) of the MP and CP was predicted (Table 2). The most common modification was serine and threonine phosphorylation at positions 35, 253 (previously described in the work by Tarquini et al. (2019) [38]), 276, 277, 281, 290, 291, 295, 300, 303, 342, 356, 363, 364, and 366 of the MP and at position 94 of the CP. In addition, we identified putative targets for N-glycosylation at positions 31, 293, and 340 and for N-myristoylation at positions 272, 279, 292, and 343.

### 2.5. Genetic Variability of GPGV and Manifestation of Symptoms

The presence of an association between GPGV genome variability and manifestation of symptoms was assessed for the Russian isolates found in this study. In Russian vineyards, the majority of isolates did not show GLMD symptoms. Phylogenetic analysis showed that symptomatic isolates are uniformly distributed throughout the tree (Supplementary Figure S2). Analysis of the MP/CP region did not show any correlation of the presence of specific SNPs with the presence of symptoms.

Two isolates, KR285 (symptomatic) and KR408 (asymptomatic), had a C to T substitution at position 6685 of GPGV, which resulted in a shortening of MP by 6 amino acids (Supplementary Figure S4). The RC309 isolate (symptomatic) had a C to T substitution at position 6688, resulting in the appearance of a stop codon 5 amino acids earlier. However, the absence of these polymorphisms in other symptomatic Russian isolates indicates that there is no correlation between the manifestation of symptoms and the presence of premature stop codons in the MP gene.

In addition, we examined the phylogenetic relationships of Russian isolates with representative isolates found on symptomatic and asymptomatic vines in previous studies [36,39] (Supplementary Figure S5). All symptomatic isolates clustered together, with one symptomatic Russian isolate (KR285) and four asymptomatic isolates (RC281, RC288, KR1495, and KR267) located next to them. In representative isolates, we identified the C/T polymorphism at position 6685 of the GPGV genome. Among the Russian isolates, only KR285 contains a premature stop codon, which is consistent with its correlation with the symptomatic group. It should be noted that KR408 and RC309 with premature stop codons in MP belonged to other clades and were asymptomatic. In addition, other isolates, RC281, RC288, KR1495, and KR267, which are close to the symptomatic clade, did not contain premature stop codons. Most of the Russian isolates clustered together and were located near asymptomatic representative isolates, which are characterized by the absence of the C/T polymorphism (at position 6685).

We also analyzed the relative titer of GPGV obtained from the qPCR results and compared it with the presence of symptoms on the grapevines (Supplementary Table S2). According to our data, GPGV symptoms were observed in plants both with high and with low relative ratios, which indicates the absence of a correlation between symptoms and GPGV titer in the Russian isolates.

## 3. Discussion

In the present study, three main viticultural regions in southern Russia (Krasnodar Krai, Stavropol Krai, Republic of Crimea) were examined for the presence of GPGV and its genetic variability. For the first time in Russia, GPGV was found in vineyards in the Republic of Crimea and the Stavropol Krai. Using the qPCR method, we detected the presence of GPGV in 74% of the analyzed samples, which reflects the widespread distribution of GPGV in southern Russia. The prevalence of GPGV in Russian vineyards is comparable to the infection rate of this virus in grapes in other countries, including Italy, at the level of 70–90% [15,36,37].

We detected GPGV not only in samples with GLMD symptoms but also in asymptomatic ones, which comprised the majority of the samples collected for the purposes of our monitoring. It should be noted that the phytosanitary condition of the grapevine plantation played a large role in the detection of GLMD symptoms. Therefore, mild symptoms may be masked due to infection with other viral and non-viral pathogens and under adverse weather and agricultural conditions [48,49]. The grapevine growing environment may also affect the manifestation of symptoms. For instance, soil type and terrain type have been found to correlate with the frequency of manifestation of symptoms [50]. Therefore, assessment of the manifestation of GPGV symptoms for Russian isolates was problematic, since most of the grapevines we examined were also affected by other viruses (Supplementary Table S2), and had symptoms of infection with phytoplasma and fungi [51,52,53,54]. Similar problems have been noted in other studies that reported the inability to link specific symptoms to the presence of GPGV given the mixed infection status of grapevines [4,37,55,56]. Moreover, an earlier assessment of GPGV titer in symptomatic and asymptomatic plants suggested the presence of a correlation between symptoms and high virus titer, thus confirming the potential role of GPGV in the development of GLMD syndrome [37,39]. However, a study by Morán et al. (2018) failed to establish a clear relationship between the presence of symptoms and GPGV titer. Here, we did not establish a correlation between the titer of Russian isolates of GPGV and the manifestation of GLMD in grapes [41]. Therefore, future studies on virus-free plants under controlled greenhouse conditions may help to establish the relationship between the presence of GLMD symptoms and the presence of GPGV in the plant. However, it is necessary to remember the limitations of the model experiment in the greenhouse and the difficulty in transferring results to vineyards, each of which has unique agrotechnical and weather conditions that affect the manifestation of symptoms in plants.

Analysis of the localization of SNPs in the genomes of Russian isolates showed their uniform distribution throughout the MP/CP region. At the same time, about 19% of polymorphisms lead to nonsynonymous mutations in the MP gene, mainly at its 3′ terminus, and 5% in the CP gene, which is expressed as a high CP identity score (99–100%). Three Russian isolates contained premature stop codons (SNPs at positions 6685 and 6688 of the GPGV genome) that were previously described in symptomatic samples [36,41]. Most of the Russian isolates did not contain these polymorphisms, which does not allow us to establish a correlation between their presence and the manifestation of symptoms.

In our study, the predicted PTMs of amino acids proteins that appeared due to non-synonymous substitutions in the MP/CP region may affect the life cycle of the virus and its pathogenicity [57]. Among 23 PTM sites, 22 were found in MP and only 1 site (a threonine phosphorylation site) in CP. This site and the 15 other phosphorylation sites found in MP may play a fundamental role in the regulation of viral infection in the plant [58]. It has previously been shown that phosphorylation of RdRp and MP amino acids is involved in the regulation of the synthesis of viral RNAs and RNA transport through the plasmodesma; it also influences the development of symptoms and the accumulation of viral RNA [59,60,61,62,63]. In addition, CP phosphorylation regulates the intercellular movement of viral particles by modulating RNA binding [64,65].

We also identified new potential sites for glycosylation and myristoylation of MP that are associated with polymorphisms. The role of these types of PTMs in the life cycle of plant viruses has not been sufficiently studied, although there is evidence suggesting that glycosylation has an important role in the life cycle of mammalian RNA viruses, namely in viral penetration, viral replication and maturation, virulence, and pathogenicity [66]. In addition, N-myristoylation can play a structural role in stabilizing the spatial structure (conformation) of proteins, and provide membrane targeting and binding [67,68]. Therefore, the PTMs of MP and CP predicted by us require further study and molecular confirmation to establish their role in the epidemiology of GPGV.

Thus, this study demonstrated a high level of GPGV distribution in Russian vineyards. Because the vineyards we examined serve as mother plantations on a number of farms, the information about the presence of GPGV in the grapevines is important for prevention of the spread of this virus to new vineyards when they are established. The absence of a clear correlation between the variability of the MP and CP genes and the manifestation of symptoms in the plant makes it necessary to conduct further research aimed at studying the biology and epidemiology of GPGV.

## 4. Materials and Methods

### 4.1. Phytosanitary Monitoring of Vineyards

Phytosanitary monitoring of grapevine plantations was carried out in the Krasnodar Krai and Stavropol Krai and in the Republic of Crimea in the summer and autumn periods from 2014 to 2020 [54]. For analysis, we collected shoots and leaves from various layers of plants with symptoms of viral diseases, including those with symptoms of GPGV (Supplementary Figure S6). Samples showing chlorotic spotting and leaf deformation, growth retardation, stunted and bushy vines, and the presence of shortened internodes and zigzag growth of shoots were considered symptomatic. For GPGV detection, we used 1347 samples: 396 (29%) samples were collected in the vineyards of the Krasnodar Krai, 918 (69%) in the Republic of Crimea, and 33 (2%) in the Stavropol Krai. The collected material was stored at +4 °C during transportation, and then the samples were stored at −20 °C.

### 4.2. GPGV Detection

Total RNA was isolated from selected grapevine samples according to the method described in Rott and Jelkmann (2001) [69]. The quality of the isolated RNA was assessed by electrophoresis in 1.2% agarose gel. For reverse transcription (RT), 2 µL of RNA, Random Hexamer as a primer, and the RevertAid H Minus Reverse Transcriptase kit (Thermo Fisher Scientific, Waltham, Massachusetts, USA) were used according to the manufacturer’s protocol. The synthesized cDNA was stored at −20 °C.

A control amplification of the 18S rRNA gene was performed with the primers 18S-H325 (AAACGGCTACCACATCCAAG) and 18S-C997 (GCGGAGTCCTAAAAGCAACA) [70]. The reaction mixture consisted of 1x Taq Buffer supplemented with (NH_4_)_2_SO_4_, 0.2 mM of each dNTP, 1 µM of each primer, 2.5 mM MgCl_2_, 0.375 U of Taq polymerase (Thermo Fisher Scientific, USA), and 1 µL of cDNA as a template. The amplification conditions were 94 °C for 3 min, followed by 35 cycles of 94 °C for 45 s, 55 °C for 45 s, 72 °C for 1 min, and a final elongation step of 72 °C for 5 min. Reverse transcription and amplification were performed on a Thermal Cycler T100 (BioRad, USA).

To determine GPGV in selected samples, real-time quantitative RT-PCR (qRT-PCR) was performed using TaqMan^®^ probes. As a result of the analysis of the literature, we chose primers and probes for the coat protein of GPGV [37], the *GAPDH* gene encoding glyceraldehyde-3-phosphate dehydrogenase [37], and the chaperonin 21 gene (*Cpn*21) from the grape chloroplast genome [71] (Supplementary Table S8).

The main qPCR parameters (efficiency, slope, R^2^, and Y-intercept) were determined in simplex (for CP, *GAPDH*, and *Cpn*21) and duplex reactions (for CP/*GAPDH* and CP/*Cpn*21). The qPCR reaction was performed in a 10 µL volume containing 1x BioMaster HS-qPCR reaction mixture (Biolabmix, Novosibirsk, Russia), 150 nM of each primer, 200 nM of probe, and 1 µL of cDNA. To construct a standard curve, we prepared a series of 3-fold dilutions of the cDNA of the control sample—by 3, 9, 27, and 81 times. Each dilution point was analyzed in three technical replicates. The values of the slope, R^2^, Y-intercept, E, and mean Cq for CP were determined using the LightCycler 96 SW1.1 software (Roche, Mannheim, Germany). To convert the indicator of instrumental efficiency, we used the formula:

(1) $$E=\left[{E^{\prime }}^{\left(\frac{1}{-S}\right)}-1\right]*100\%,$$

where *E* is the efficiency of qPCR, *E*′ is the indicator of the instrumental efficiency, and *S* is the slope of the standard straight line [72]. Based on the obtained qPCR parameters, we chose the optimal duplex for further detection of GPGV.

Detection of GPGV in the samples was conducted in two technical replicates. Each experiment included a negative control without template and a positive control with cDNAs from a GPGV-infected plant. Amplification was carried out using a LightCycler 96 (Roche, Mannheim, Germany) under the following PCR conditions: 5 min at 95 °C; 15 s at 95 °C, 60 s at 60 °C (7 cycles without detection of fluorescence signal); 15 s at 95 °C, 60 s at 60 °C (43 cycles with detection of fluorescence signal). Samples were considered positive if the value of Cq did not exceed 40, and negative if the value of Cq was absent or greater than 40. To determine the relative titer of GPGV, the following formula was used:

(2) $$Ratio=\frac{E{’}_{R}^{Cq_{R}}}{E{’}_{T}^{Cq_{T}}},$$

where *Ratio* is a relative dimensionless number that is meaningful only when comparing samples with each other and characterizes the relative titer of GPGV, $E{’}_{T}$ is the amplification efficiency of the target gene, $E{’}_{R}$ is the amplification efficiency of the internal control gene, $Cq_{T}$ is the quantification cycle of the target gene, and $Cq_{R}$ is the quantification cycle of the internal control gene [73].

### 4.3. Variability of GPGV and Phylogenetic Analysis

The genetic diversity of GPGV was studied in relation to the MP and CP genes on 119 GPGV-positive samples with a relative ratio of more than 7 × 10^−3^. No more than one sample from each grapevine plantation was used for analysis (Supplementary Table S2). The selection of primers for MP/CP amplification was carried out for conservative regions of 464 sequences of GPGV isolates (available from the GenBank as of July 2020) [74]. Alignment was performed using the MEGA X software [75]. The specificity of the selected primers (Supplementary Table S9) was verified in silico using the blastn tool in NCBI. PCR was performed in a 15 µL volume containing 1× Phusion HF buffer, 1 µL of cDNA, 0.2 mM of each dNTP, 1.5 µM of each primer, and 0.3 U of Phusion Hot Start II High-Fidelity DNA Polymerase (Thermo Fisher Scientific, USA). The following cycling conditions were used: initial denaturation at 95 °C for 5 min, followed by 39 cycles of 98 °C for 10 s, 56 °C for 30 s, and 72 °C for 20 s, and a final extension at 72 °C for 10 min. The amplification products were isolated from the agarose gel using the Cleanup Standard kit (Evrogen, Moscow, Russia) according to the manufacturer’s protocol. Each amplicon was sequenced in both directions by the Sanger method using the BigDyeTM Terminator v3.1 Cycle Sequencing Kit (Thermo Fisher Scientific, USA) on an ABI PRIZM 3730 automatic sequencer according to the manufacturer’s instructions.

The resulting sequences were analyzed using the Finch TV 1.4.0 software [76] and MEGA X software [75] and deposited in the GenBank.

The existence of recombination events in the nucleotide sequences of the MP/CP region of Russian GPGV isolates was analyzed by the Recombination Detection Program v4.101 (RDP4) with the default settings for the methods RDP, GENECONV, BOOTSCAN, MAXCHI, CHIMAERA, SISCAN, and 3SEQ [77]. Recombination breakpoints were considered significant events if identified by four or more methods.

For the resulting MP/CP nucleotide sequences, we determined the pairwise identity (%) with the closest isolate from the NCBI database. The comparison of Russian GPGV isolates among themselves was performed with the Sequence Demarcation Tool (SDT v1.2) using the Clustal W alignment algorithm [78]. The pairwise matrices were aligned for the MP/CP nucleotide sequences with the length of 1600 bp (starting from 5578 bp to 7177 bp in NC_015782.2), and for the amino acid sequences of MP and CP. The frequency of identity scores was calculated as the number of identity scores of each value from pairwise identity matrices (Supplementary Tables S4, S6 and S7) divided by the total number of identity scores expressed as a percentage.

Phylogenetic analysis involved 119 Russian isolates and 623 global isolates represented by the complete GPGV genomes and MP/CP gene sequences available from the GenBank (as of February 2022). To establish a relationship between the manifestation of symptoms and the presence of polymorphisms in the MP/CP region, we carried out a phylogenetic analysis using the sequences of Russian isolates with a length of 1600 bp and representative isolates described previously [36,39]. Multiple alignment was performed using the MEGA X software [75]. Phylogenetic trees were generated using the maximum likelihood (ML) method based on the Tamura–Nei model [79] with 1000 bootstrap replicates. 

The presence of polymorphisms in the MP/CP region was analyzed using the Bioedit v7.2.5 software, setting the shade threshold to 100% [80]. The variability of different nucleotide residues of the MP and CP genes of GPGV was confirmed using the entropy plot function (H(x)) implemented in the BioEdit 7.2.5 software. Virus ORFs in 1600 nucleotide bp sequences were predicted using the ORFfinder tool from NCBI with a minimum length of 50 amino acids and ATG as the start codon [81]. The presence of possible PTMs was analyzed using the ScanProsite program, with the option of running the scan at high sensitivity [82].

## Figures and Tables

**Table 1:** Calibration curve parameters of the simplex and duplex qPCR assays.

Assay	Gene	PCR Efficiency, %	Slope	R^2^	Y-Intercept
Simplex	CP	96	−3.4114	0.97	24.75
	*Cpn*21	102	−3.2668	0.98	21.33
	*GAPDH*	74	−4.1485	0.98	19.20
Duplex	CP	97	−3.4034	0.99	24.64
	*Cpn*21	94	−3.4856	0.97	22.85
Duplex	CP	93	−3.4939	0.97	22.84
	*GAPDH*	78	−4.0060	0.98	18.63

**Table 2:** List of the polymorphic sites of MP/CP Russian GPGV isolates.

Gene	Position in Genome	Position in Protein	Codon	Amino acid	Occurrence of aa	Variant Frequency, %	Putative Post-Translational Modification
MP	5669	31	AAT	N	118	99.2	N-glycosylation
			AGT	S	1	0.8	- *
	5681	35	TCG/TCA	S	113	95.0	Protein kinase C phosphorylation
			TTG	L	6	5.0	-
	6335	253	AGC/AGT	S	80	67.2	Protein kinase C phosphorylation
			AAC/AAT	N	37	31.1	-
			ARC	X	2	1.7	-
	6392	272	GGA/GGR	G	116	97.5	N-myristoylation
			GAA	E	3	2.5	-
	6404	276	TTC/TTY	F	118	99.2	-
			TCC	S	1	0.8	Casein kinase II phosphorylation site
	6407	277	CTT	L	24	20.2	-
			TTT	F	88	73.9	-
			TCT	S	6	5.0	Casein kinase II phosphorylation site
			ATT	I	1	0.8	-
	6413	279	GAA/GAG/GAR	E	118	99.2	-
			GGA	G	1	0.8	N-myristoylation
	6419	281	ACA	T	115	96.6	Casein kinase II phosphorylation
			GCA	A	3	2.5	-
			ATA	I	1	0.8	-
	6446	290	CGC/CGA/AGA/CGT	R	115	96.6	-
			TGC	C	1	0.8	-
			MGC	X	2	1.7	-
			AGC	S	1	0.8	Protein kinase C phosphorylation
	6449	291	ACT/ACC	T	112	94.1	Casein kinase II phosphorylation
			ATT	I	2	1.7	-
			AAT	N	1	0.8	-
			GCC/GCT	A	3	2.5	-
			RCT	X	1	0.8	-
	6452	292	GAA/GAG	E	113	95.0	-
			GGA	G	1	0.8	N-myristoylation
			RAA	X	2	1.7	-
			GCA	A	1	0.8	-
			AAA	K	2	1.7	-
	6455	293	AAT	N	112	94.1	N-glycosylation
			AGT/AGC	S	3	2.5	-
			AAA	K	1	0.8	-
			GAT	D	3	2.5	-
	6461	295	TCA/TCG	S	116	97.5	Protein kinase C phosphorylation
			YCA	X	1	0.8	-
			CCA	P	2	1.7	-
	6476	300	TTC/TTT	F	106	89.1	-
			TCC	S	8	6.7	Protein kinase C phosphorylation
			TTA/CTC	L	4	3.4	-
			TTM	X	1	0.8	-
	6485	303	GGT	G	116	97.5	-
			RGT	X	1	0.8	-
			GAT	D	1	0.8	-
			AGT	S	1	0.8	Casein kinase II phosphorylation
	6596	340	GAT	D	116	97.5	-
			AAT	N	3	2.5	N-glycosylation
	6602	342	TCA	S	117	98.3	Casein kinase II phosphorylation
			CCA	P	2	1.7	-
	6605	343	GGA	G	118	99.2	N-myristoylation
			AGA	R	1	0.8	-
	6644	356	GTT	V	3	2.5	-
			GCT	A	111	93.3	-
			ACT	T	3	2.5	Protein kinase C phosphorylation
			GVT/RCT	X	2	1.7	-
	6665	363	ACT	T	113	95.0	Protein kinase C phosphorylation
			GCT	A	5	4.2	-
			RCT	X	1	0.8	-
	6668	364	TCA	S	118	99.2	Casein kinase II phosphorylation
			CCA	P	1	0.8	-
	6674	366	GCT	A	3	2.5	-
			GTT	V	111	93.3	-
			ATT	I	2	1.7	-
			ACT	T	2	1.7	Protein kinase C phosphorylation
			GYT	X	1	0.8	-
	6686	370	TAA	Stop codon	2	1.7	-
			CAA	Q	116	97.5	-
			YAA	X	1	0.8	-
	6689	371	TAA	Stop codon	1	0.9	-
			CAA	Q	115	98.3	-
			YAA	X	1	0.9	-
	6701	376	TGA	Stop codon	116	100.0	-
CP	6870	94	ACT/ACC/ACY	T	118	99.2	Casein kinase II phosphorylation
			ATT	I	1	0.8	-

*—No sites.

## Data Availability

Representative sequences were deposited in GenBank under the accession numbers: OM925754, OM973969-OM973988, ON013445-ON013499, ON013518-ON013545, ON033799-ON033813.

## Supplementary Materials

The following supporting information can be downloaded at: https://www.mdpi.com/article/10.3390/plants11081061/s1, Figure S1: Color-coded pairwise identity matrix performed for Russian GPGV isolates genomes for nucleotide MP/CP sequences, movement proteins (MPs), and coat proteins (CPs); Figure S2: Phylogenetic analysis of Russian isolates of GPGV based on the nucleotide sequences of the movement protein (MP) and coat protein (CP) genes; Figure S3: Graphical representation of the movement protein (MP) and coat protein (CP) genes generated using the entropy plot function (H(x)); Figure S4: Alignment of MP/CP nucleotide sequences and movement proteins (MP) of Russian isolates; Figure S5: Phylogenetic analysis of Russian and representative Italian GPGV isolates [36,39]; Figure S6: Symptoms of grapevine leaf mottling and deformation disease observed on Vitis vinifera; Table S1: Quantification cycle (Cq) values of qPCR for the GPGV coat protein gene in simplex and duplex with different internal control genes; Table S2: Basic information of the sampled vineyards and qPCR parameters; Table S3: Amplified sequences of viruses uploaded into GenBank with their identifier; Table S4: The pairwise identity matrix of nucleotide sequences among Russian isolates; Table S5: Frequency of the identity scores for nucleotide MP/CP sequences, movement proteins (MPs), and coat proteins (CPs) of Russian GPGV isolates; Table S6: The pairwise identity matrix of movement proteins’ amino acid sequences among Russian isolates; Table S7: The pairwise identity matrix of coat proteins’ amino acid sequences among Russian isolates; Table S8: List of primers and probes used for qPCR; Table S9: List of primers used for phylogenetic analysis.
