# Supplementary material for: Occurrence and Genetic Characterization of Grapevine Pinot Gris Virus in Russia
Source: Plants (Basel). 2022 Apr 13;11(8):1061. doi: 10.3390/plants11081061 (PMC9028157; doi:10.3390/plants11081061)
Supplement: Supplementary file 1 [file plants-11-01061-s001.zip › Supplementary Figures.pdf]

Occurrence and genetic characterization of Grapevine Pinot gris virus in Russia

Darya Shvets [1], Svetlana Vinogradova [1,*]

[1]Institute of Bioengineering, Research Center of Biotechnology of the Russian Academy of Sciences, Leninsky Prospect 33, 119071 Moscow, Russia; darya-shv@mail.ru (D.S.); coatprotein@bk.ru (S.V.)

[*]Correspondence: coatprotein@bk.ru

Supplementary materials

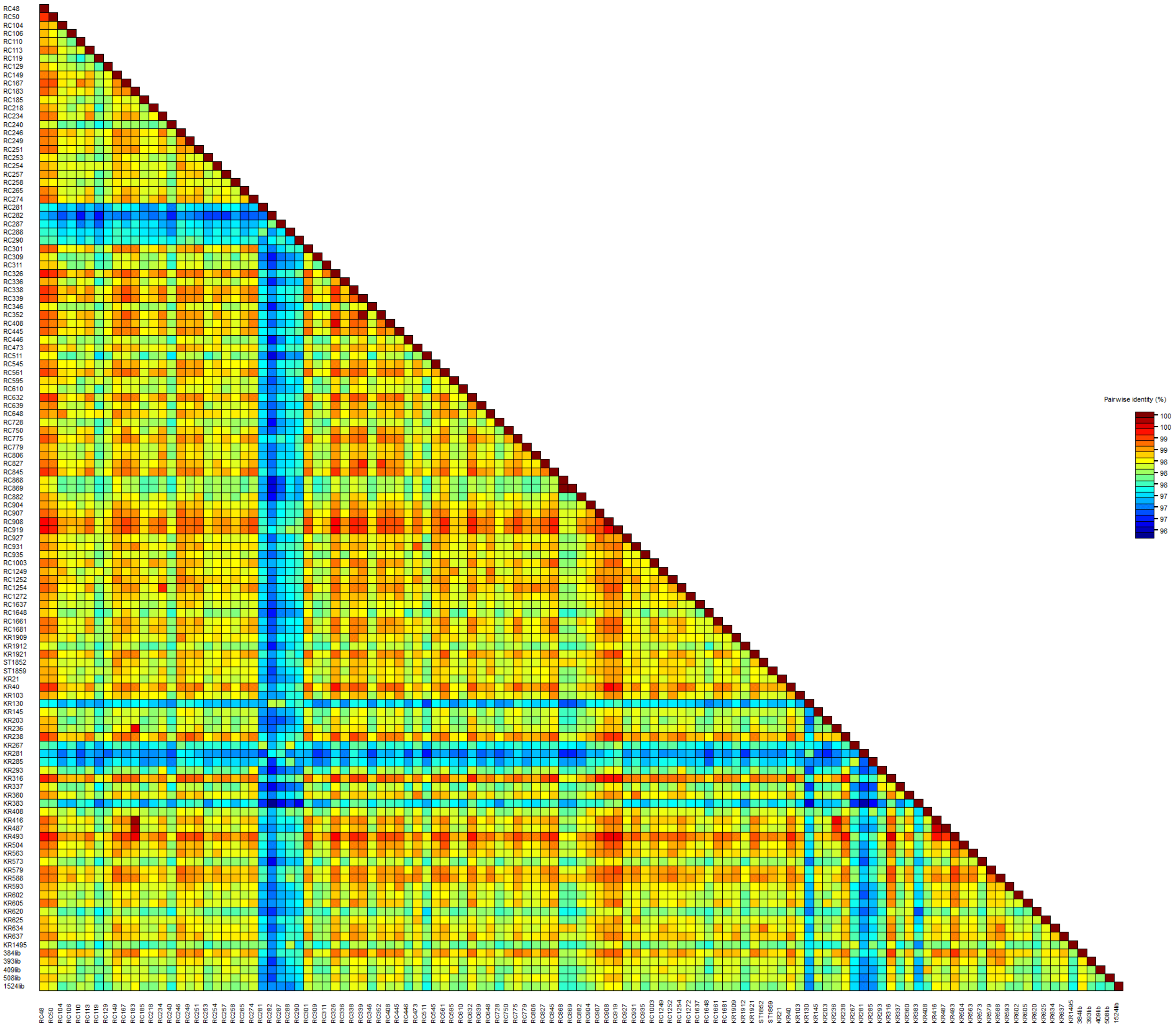

(**a**)

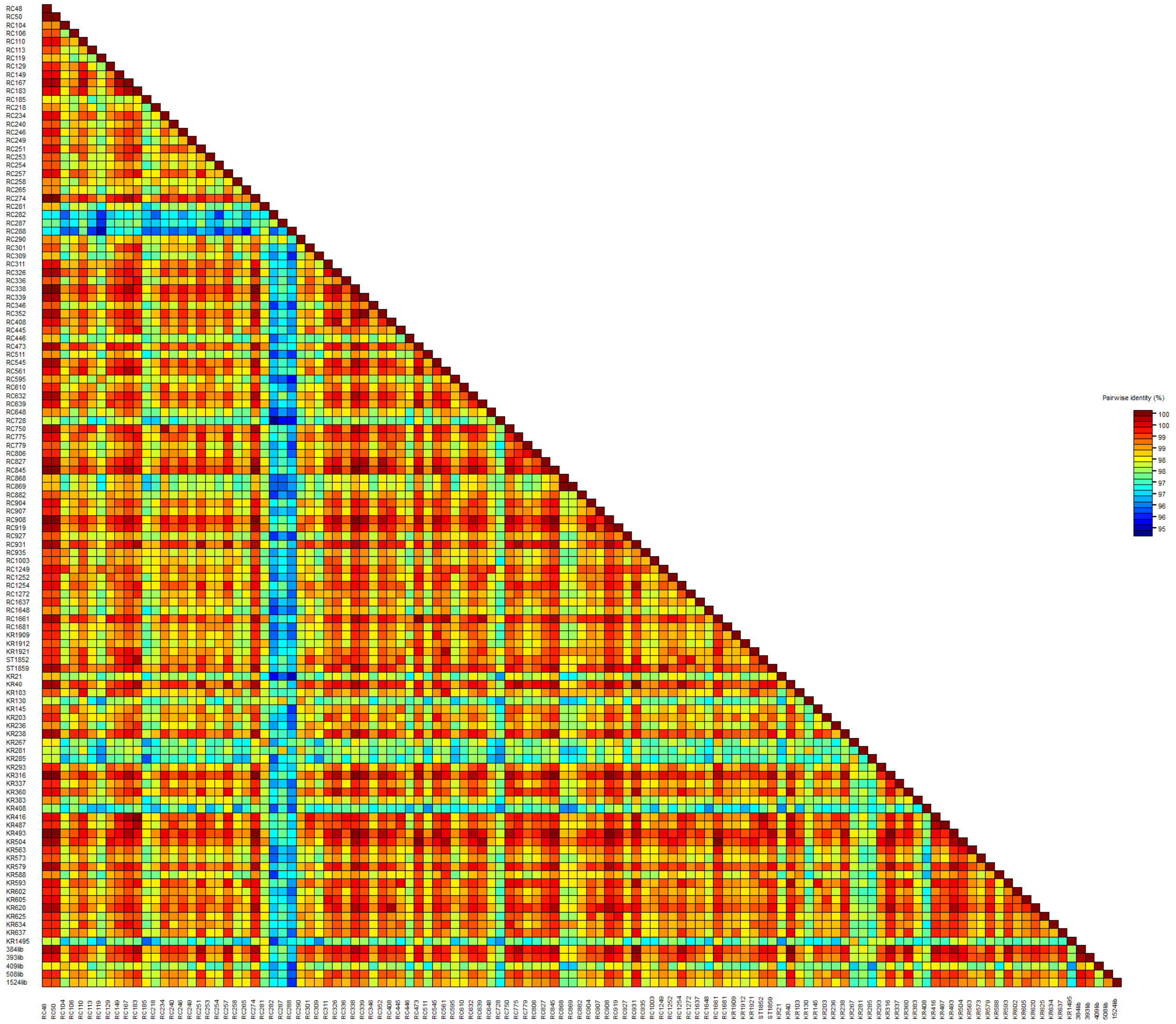

(**b**)

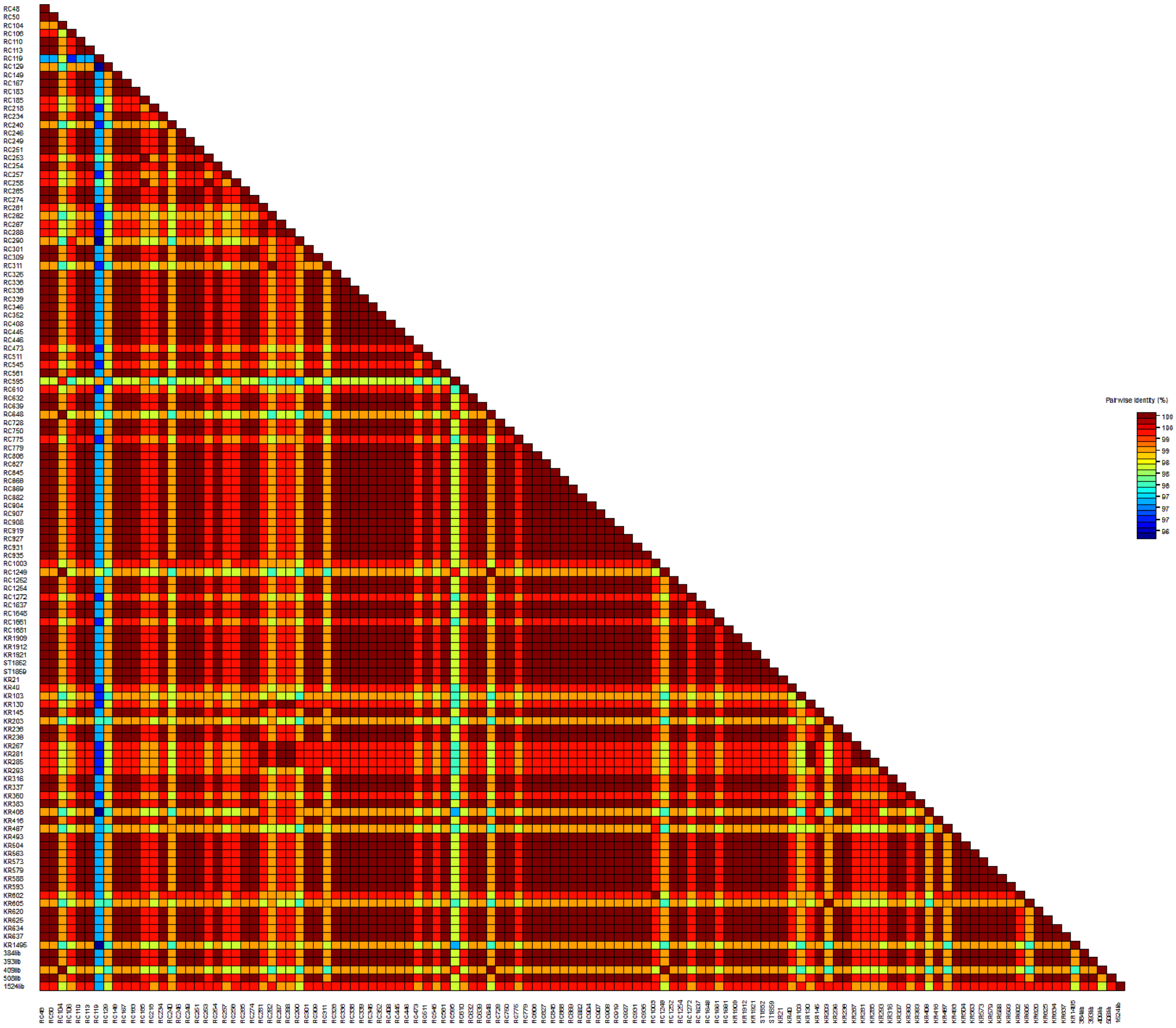

(**c**)

Figure S1 (a-c). Colour-coded pairwise identity matrix performed from Russian GPGV isolates genomes for (**a**) nucleotide MP/CP sequences, (**b**) movement proteins (MP) and (**c**) coat proteins (CP). GenBank accession numbers for each isolate are presented in Supplementary Table 3.

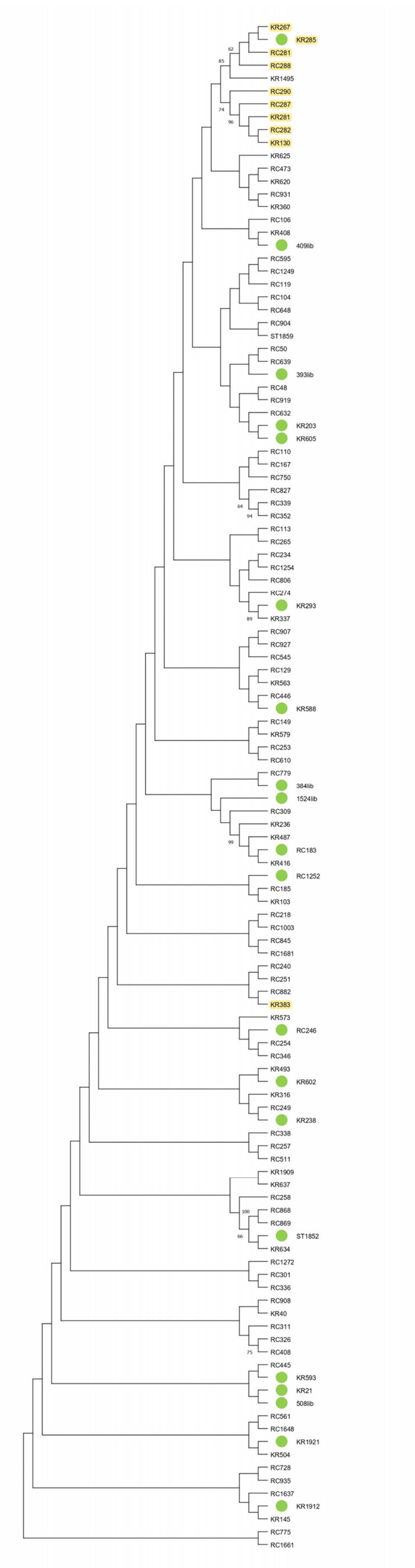

Figure S2. Phylogenetic analysis of Russian isolates of GPGV based on nucleotide sequences of the movement protein (MP) and coat protein (CP) genes. The symptomatic isolates obtained in this study are indicated by the “green mark”. Isolates from the Krasnodar region, Stavropol region and the Republic of Crimea are marked as KR, ST and RC, respectively. Divergent insolates are highlighted in yellow. The tree was constructed using the Maximum Likelihood method. The numbers at the nodes indicate bootstrap support (1,000 replicates); values above 60% are shown.

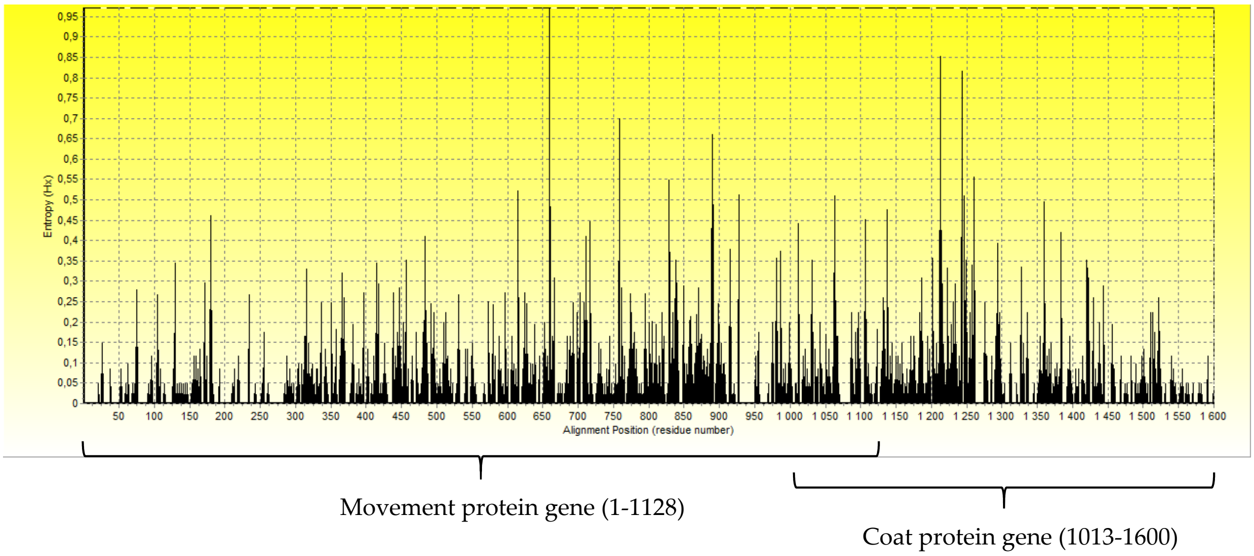

Figure S3. Graphical representation of the movement protein (MP) and coat protein (CP) genes generated using the entropy plot function (H(x)). The plot shows the variability of the different nucleotide residues in alignment with Russian isolates of GPGV.

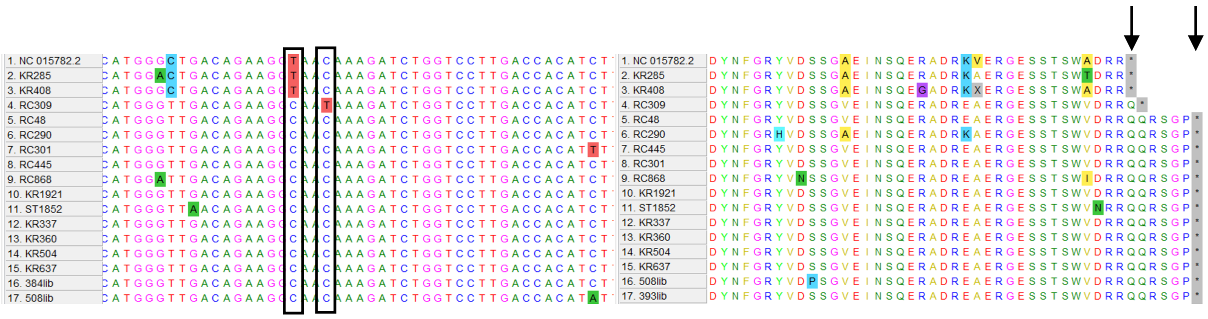

(**a**) (**b**)

Figure S4 (a, b). Alignment of (**a**) MP/CP nucleotide sequences; (**b**) movement proteins (MP) of Russian isolates. 6685 and 6688 T/C polymorphisms are marked in boxes. The stop codons are indicated by gray stars.

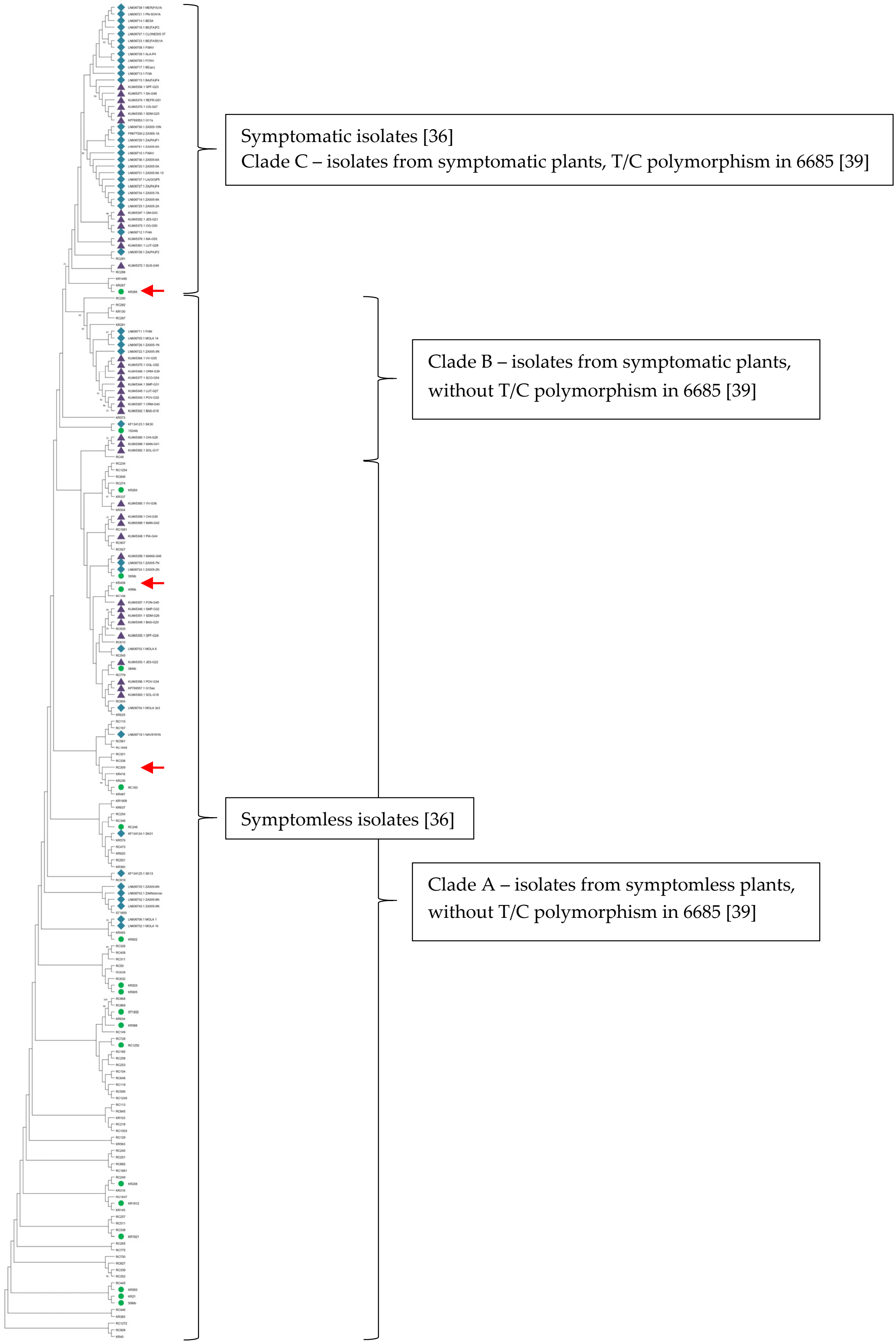

Figure S5. Phylogenetic analysis of Russian and representative Italian GPGV isolates. Representative Italian GPGV isolates from Saldarelli et al. (2015) [36] are marked as a blue diamond; from Bertazzon et al. (2017) [39] are marked as a purple triangle; symptomatic Russian isolates are marked as a green circle. Isolates with early stop codons are marked with a red arrow. The tree was constructed using the Maximum Likelihood method. The numbers at the nodes indicate bootstrap support (1,000 replicates); values above 60% are shown.

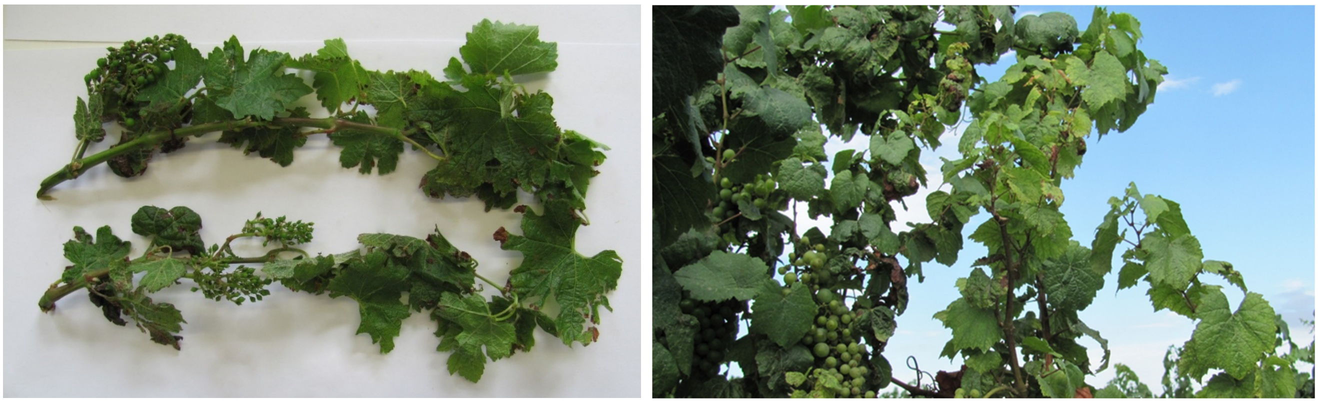

(a) **(b)**

Figure S6 (a, b). Symptoms of grapevine leaf mottling and deformation disease (decline, short internodes, uneven size of berries and leaf deformation) observed on *Vitis vinifera* cv. (a) Muscat Golodrigi and (b) Rkatsiteli infected with GPGV.
